# Cross-National Trends in Early Sexual Initiation Among 15-Year-Old Adolescents, 2002–2022

**DOI:** 10.3389/ijph.2025.1607711

**Published:** 2025-03-11

**Authors:** András Költő, Kristina Winter, Rachael Maloney, Louise Lunney, Christiana Nicolaou, Alina Cosma, Margreet de Looze, Colette Kelly, Gina Martin

**Affiliations:** ^1^ Health Promotion Research Centre, University of Galway, Galway, Ireland; ^2^ Institute of Medical Sociology, University Hospital in Halle, Halle, Bavaria, Germany; ^3^ Institute for Social Medicine, Rehabilitation Sciences and Health Services Research, Hochschule Nordhausen, Nordhausen, Thuringia, Germany; ^4^ Centre for Educational Research and Evaluation, Ministry of Education and Culture, Nicosia, Cyprus; ^5^ School of Psychology, Trinity College Dublin, Dublin, Ireland; ^6^ Olomouc University Social Health Institute (OUSHI), Olomouc, Czechia; ^7^ Department of Interdisciplinary Social Science, Faculty of Social and Behavioural Sciences, Utrecht University, Utrecht, Netherlands; ^8^ Faculty of Health Disciplines, Athabasca University, Athabasca, AB, Canada

**Keywords:** adolescents, sexual health, early sexual initiation, HBSC study, trends

## Abstract

**Objectives:**

We examined how the proportion of adolescents who engaged in early sexual intercourse (before the age of 14) changed between 2002 and 2022 across 37 countries.

**Methods:**

Data of 15-year-old adolescents participating in the 2002, 2006, 2010, 2014, 2018, and 2022 survey rounds of the Health Behaviour in School-aged Children study were analysed (*N* = 312,702). We used uni- and multivariate multilevel binary logistic regression models to test whether rates of early sexual initiation changed over time. Country, gender, family affluence, parental support and the clustering effect of school were incorporated in the statistical models; linearity was tested by cubic and quadratic terms in the multivariate models.

**Results:**

A significant but very small decline over time was found in early sexual initiation across survey years. Girls and less affluent adolescents had lower odds of early initiation. Parental support (and its interaction with time) also had a significant but small protective role.

**Conclusion:**

Despite a small decrease over time, still 4% of participants reported early sexual initiation in 2022. Concerted and sustained efforts are needed to support adolescent sexual health.

## Introduction

Many individuals have their first romantic and sexual experiences during adolescence [1–3]. Sexual development is a normative part of the transition to adulthood [4, 5]. Romantic and sexual relations can positively contribute to young people’s lives, for example, through positive social relationships or increased self-esteem. *Early* sexual initiation, on the other hand, is associated with risks, including decreased likelihood of condom and contraceptive use; sexually transmitted infections; psychosocial health problems; substance use; unplanned pregnancy and unsafe abortion ([4, 6–17], systematic reviews: [18–20]). These may partly be attributed to that early sexual initiation is often non-consensual and is more often regretted afterwards than non-early initiation [21, 22].

“Early” sexual initiation is defined by varied age thresholds [4, 12, 23], possibly because what is considered “early” partly depends on cultural norms and social perceptions. Some define it as being younger than 17, 16 or 15 years [4, 10, 23–26], or even under 13 years at the first sexual intercourse [27–29]. Younger than 14 is the threshold often used in the Health Behaviour in School-aged Children (HBSC), a World Health Organization collaborative cross-cultural study (e.g., [10, 30]).

Views on “appropriate” age for first sexual initiation also vary [21, 26], partly due to societal attitudes towards adolescent sexual behaviour and access to sexual education and healthcare services. Socio-economic, biological and social mechanisms also contribute to the complexity of sexual initiation [10, 31–33]. Socio-cultural rules and gender scripts defining the normative age for first sexual intercourse also show large cross-cultural variation [11, 26]. Girls in many cultures face double standards regarding sexuality: compared to boys, they are more often pressured into having sex but are considered immoral if they do so [21, 34–38].

The most recent trends analysis in this area [21] used data from the 2010, 2014, and 2018 rounds of HBSC, to map how sexual initiation among 15-year-olds across 33 countries changed over time. In many countries, an overall decrease was found. More boys than girls had reported sexual initiation, although the gender gap slightly decreased between 2010 and 2018. Earlier studies found an inconclusive, mostly stable trend between 2002 and 2010 in both reported sexual initiation and early initiation among 15-year-olds [30]. A long-term trend study, analysing the changes in early sexual initiation in the last two decades until recent years, is still missing from the literature. There is evidence that adolescent sexual behaviour sometimes evolves in a non-linear way over time [39], as it is influenced by a variety of factors that do not act in a consistent, linear manner. For example, a sudden shift in societal, cultural or political attitudes, the implementation of public health campaigns or social distancing measures – like the ones introduced in response to the COVID-19 pandemic – may result in a sudden change in sexual initiation, which would be missed if only linear trends were considered.

Evidence is mixed on how socio-economic status (SES) of adolescents’ families impact the timing of their sexual initiation. A meta-analysis concluded that adolescents from low-SES families are more likely to report early sexual initiation [19], although a Serbian study found no association [40]. In 13 of the 42 countries where data was collected on sexual behaviours of adolescents in the 2022 HBSC survey round, boys from low-affluent families were more likely to report sexual intercourse than their peers from high-affluent families; a similar effect was found among girls in seven countries [41]. While earlier trend analyses of HBSC data on sexual initiation did not account for the potential impact of SES, these findings highlight the importance of investigating its role in early sexual initiation.

The role of parental support and communication between adolescents and parents also need to be considered. Worldwide analysis indicates that parental monitoring is protective against early sexual intercourse, but parental support is not [26]. Most single-country cross-sectional and longitudinal studies [19, 42], on the other hand, show that good adolescent–parent relationships delay sexual initiation. A warm and supportive parental figure, with whom the adolescent can easily discuss their problems, might promote the adolescent’s self-esteem, which is protective against early sexual intercourse [43]. Adolescents are also more likely to adopt parents’ moral values and attitudes towards early sexual initiation if there is an emotional bond between them and their parents [44]. Further cross-cultural evidence is needed to confirm that parental support is protective against early sexual initiation. Since studies show that perceived quality of communication with parents increased in the last decades [45, 46], the question also emerges as to whether there is an interplay between parental communication and time in influencing early sexual intercourse.

To address these knowledge gaps, the current study raised the following research questions:

1. a) Internationally, has the proportion of adolescents engaging in early sexual initiation changed between 2002 and 2022?
  b) If there was a significant change, was it linear or nonlinear?
2. Do time trends in early initiation vary across countries?
3. Does controlling for gender and family affluence impact the trends in early sexual initiation?
4. a) Does controlling for having a supportive parental figure impact the trends in early sexual initiation?
  b) Does the relationship between parental support and trends of early sexual initiation change over time (i.e., does early initiation interact with survey year)?

## Methods

HBSC is a cross-national survey conducted every 4 years since 1983 to monitor the health and wellbeing of adolescents across Europe, Central Asia and North America. In every survey round, the HBSC employs a standardised research protocol with each country collecting data from a nationally representative sample of 11-, 13-, and 15-year-olds [47]. Stratified random cluster sampling is used, with classes nested within schools serving as the primary sampling units. Adolescents complete anonymous questionnaires in classroom settings. These questionnaires are translated from English into national languages, following a validated protocol including back-translation checks. Ethical consent was obtained from relevant institutions in each participating country.

### Sample

The present study used data from the 2002, 2006, 2010, 2014, 2018 and 2022 HBSC survey rounds. Participating countries were eligible for inclusion in the present analyses if they had collected data on both sexual intercourse and the age of first sexual intercourse at least in the last three survey rounds. The questions on sexual behaviour were only administered to 15-year-olds, therefore our analyses were limited to this age group. The analytic sample included 312,702 participants across 37 countries (51.9% girls, mean age: 15.08, SD = 0.43).

### Measures

*Early sexual initiation*: Participants were asked if they had ever had sexual intercourse; those responding “yes” were subsequently asked how old they were when they had their first sexual intercourse. The response options were “11 years old or younger”/“12”/“13”/“14”/“15”/“16 years old or older”; in some countries, “17 years old or older” was the last option. While these are not relevant to 15-year-old participants, in some survey years and countries older adolescents, aged 16–18 were also participating in the study, hence the last two response options. We dichotomised the responses into having had early (before the age of 14) or not early (at the age of 14 or older) first sexual intercourse.

A nominal variable was created from *country/region*. Participants’ *gender* was assessed by asking whether they are a boy or a girl; 845 participants (0.3%) did not answer this item [48]. They were retained for aggregated analyses but excluded from gender-disaggregated models. *Family affluence* – as an indicator of SES – was assessed using the Family Affluence Scale (FAS). This is a composite score consisting of six items (e.g., whether participants have their own bedroom, or how many cars are owned by their family) [49]. The sum score was transformed into relative groups, classifying the lowest 20%, the middle 60% and the highest 20% affluent families in each country [50]. In order to prevent further reduction of the analytic sample, we created a fourth category, consisting of participants with missing responses on the FAS.

*Survey year*: In univariate analyses encompassing all survey rounds (Steps 1–4 and 5a–8a in Table 1), we have created five dummy variables that had two values for each two consecutive survey rounds (i.e. 2002 = 0, 2006 = 1; 2006 = 0, 2010 = 1; and so forth), to account for potential fluctuations across survey rounds. In multivariate analyses including all years (Steps 5a–10a in Table 1), survey years were centred to support examination of non-linear trends. In analyses covering only the last three survey rounds (Steps 5b–10b in Table 1), we have treated survey years as categorical variables, and used 2014 as the reference.

**TABLE 1:** Analytic strategy to map trends in early sexual intercourse (Health Behaviour of School-aged Children study, 2002–2022).

Steps of analysis		Research question(s)	Relevant results presented in:
**Univariate analysis – whole dataset**			
1) Descriptive statistics for the aggregate sample: percentages of early sex, across countries and survey rounds		RQ1(a). Internationally, has the proportion of adolescents engaging in early sexual initiation changed between 2002 and 2022? RQ2. Do time trends in early initiation vary across countries?	Table 2 Figures 1, 2
2) Descriptive statistics disaggregated for gender		RQ3. Does controlling for gender and family affluence impact the trends in early sexual initiation?	Table 2
3) Univariate binary logistic regression models for the aggregate sample: odds of having early sex, compared to the previous survey round, across countries and survey rounds		RQ1(a)	Supplementary Table S4
4) Univariate binary logistic regression models disaggregated for gender		RQ3. Does controlling for gender and family affluence impact the trends in early sexual initiation?	Supplementary Table S5 (boys) and Supplementary Table S6 (girls)
**Multivariate analysis**			
**(a) Data available from every survey round (24 countries)**	**(b) Data from the last three survey rounds (37 countries)**		
5(a) Adding clustering effect	5(b) Adding clustering effect	RQ2	Arm (a): Table 3; arm (b): Table 4
6(a) Centering year and comparing linear, quadratic and cubic solutions	6(b) Treat year as categorical	RQ1(a) RQ1(b). If there was a significant change, was it linear or nonlinear?	Arm (a): Table 3; arm (b): Table 4
7(a) Adding gender and FAS as covariate	7(b) Adding gender and FAS as covariate	RQ3	Arm (a): Table 3; arm (b): Table 4
8(a) Adding supportive parental figure as a covariate	8(b) Adding supportive parental figure as a covariate	RQ4(a). Does controlling for having a supportive parental figure impact the trends in early sexual initiation?	Arm (a): Table 3; arm (b): Table 4
	9(b) Adding supportive parental figure × survey round interaction parameter as a covariate	RQ4(b). Does the relationship between parental support and trends of early sexual initiation change over time (i.e., does early initiation interact with survey year)?	Arm (a): Table 3; arm (b): Table 4
	10(b) Run Model 8(b) disaggregated by gender	RQ3	Arm (a): Table 3; arm (b): Table 4

The *quality of adolescent–parent communication* was measured by four items, tapping into how easy it is for the participant to talk to their father, mother, stepfather or stepmother about the things that really bother them. For each four items, response options were “Very easy”/“Easy”/“Difficult”/“Very difficult”/“Don’t have or see this person.” We merged the four items into a variable with having at least one parental figure with whom communication is very easy or easy = 1; not having any such parental figure = 0.

In line with the stratified sampling strategy of HBSC (classrooms clustered in schools within countries), a *school variable* was created which accounts for the potential effects of school-level norms and other school-level sources of variations in early sex not accounted for in the models. Schools were nested within countries.

### Analytic Strategy

**FIGURE 1:**
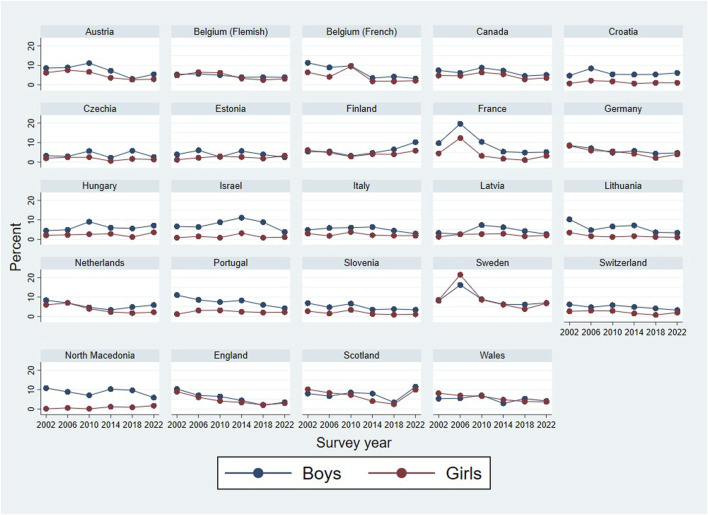
Percentage of early sexual intercourse among 15-year-olds in those 24 countries where data was available for all six survey rounds (Health Behaviour of School-aged Children study, 2002–2022).

**FIGURE 2:**
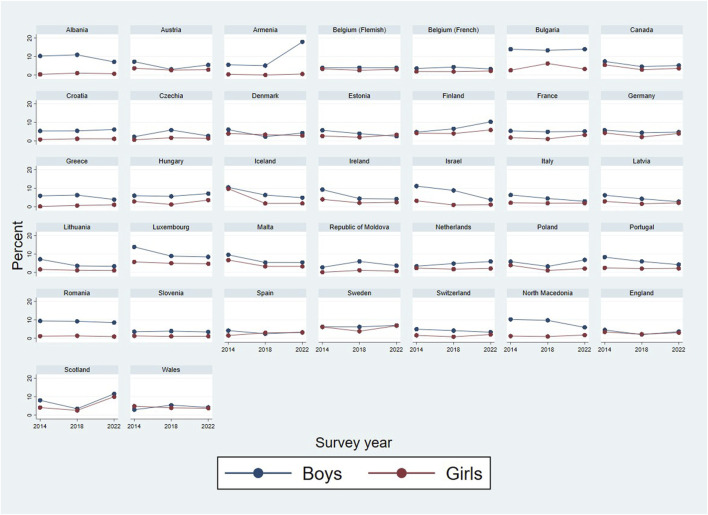
Percentage of early sexual intercourse among 15-year-olds in all 37 countries for the last three survey rounds (Health Behaviour of School-aged Children study, 2014–2022).

Steps of the analytic strategy are presented in Table 1. First, percentages of those who reported early sexual initiation were calculated, across countries and survey rounds. Chi-square tests were conducted to test the association between prevalence of early sexual initiation and survey years, in the aggregated (Model 1) and gender-disaggregated (Model 2) samples. Cramer’s *V* effect sizes for Chi-square tests were interpreted as: *V* ≤ 0.05 very small, *V* between 0.06 and 0.19 small, *V* between 0.20 and 0.29 medium, and *V* ≥ 0.30 large [51]. We used visual inspection of graphs depicting the temporal dynamics of changes across countries and genders (Figures 1, 2) to infer cross-country patterns of trends.

Next, univariate binary logistic regression models estimated participants’ odds for early sexual intercourse from the previous to the subsequent survey round, covering all survey rounds in which the given country collected data. The reference was always the previous survey round. Again, these steps were carried out for the aggregated sample (Model 3) and then separately for boys and girls (Model 4).

Data were not available from all survey years in each country. Therefore we have carried out the next steps separately for two “arms”: (a) those 24 countries where data was available for all survey rounds; (b) for all countries, covering the last three survey rounds. First, for both arms we created null models (no prediction) accounting for potential clustering effect schools nested in countries (Models 5a/5b). Following that, for arm (a), we centred years and added three separate regression terms. In addition to a linear regression term, we tested if adding a quadratic term (modelling a parabolic curve) and a cubic term (modelling trends that may increase or decrease at varying rates) results in increased model fit (Model 6a). Next, we added gender and family affluence as covariates (Model 7a); and added having a supportive parental figure as a covariate (Model 8a). For arm (b), we added year as categorical variables (comparing change from 2014 to 2018 and 2014 to 2022, respectively) (Model 6b); added gender and family affluence (Model 7b); added having a supportive parental figure (Model 8b), then tested the potential interactions of the second and third survey year and having a supportive parental figure, to account for the potential changes in parental support from 2014 to 2018 and 2014 to 2022 (Model 9b). Since adding these interaction effects reduced model fit, we stepped back and re-ran Model 8b separately for boys and girls (Model 10b). Multicollinearity was tested and the improvement of the models were compared using Akaike (AIC) and Bayesian Information Criterion (BIC).

Where countries provided a weight variable (correcting for gender, regional or socio-economic imbalances in the sample), we applied it in Steps 3–10. In the absence of actual weights, weight was set at one. Steps 1–4 were conducted in SPSS Version 28; Steps 5–10 in Stata.

## Results

### Univariate Analysis

Table 2 presents the characteristics of the analytic sample are presented. Figure 1 shows the rates (in unweighted percentages) of early sexual intercourse for 24 countries between 2002 and 2022; Figure 2 shows the rates for all 37 countries between 2014 and 2022. Respective percentages and Chi-square tests showing how the rates of early sexual intercourse are associated with survey year are presented in Supplementary Table S1 for the overall sample; Supplementary Table S2 for boys and Supplementary Table S3 for girls. The prevalence of early sexual intercourse was highest in 2006 (7.3%); and lowest in 2018 (3.7%) and 2022 (3.8%). The figures and the tables show that there was a variation across countries (and sometimes gender) in the temporal dynamics in early intercourse, albeit the effects were mostly small or very small.

**TABLE 2:** Sample characteristics, overall and across survey years (*N* = 312,702); unweighted percentages (Health Behaviour of School-aged Children study, 2002–2022).

	Total sample		2002		2006		2010		2014		2018		2022		Association with survey year
	*n*	%	*N*	%	*n*	%	*n*	%	*N*	%	*n*	%	*n*	%	
Total sample per survey round	312,702	100	31,119	10.0	42,534	13.6	52,983	16.9	58,020	18.6	57,468	18.4	70,498	22.5	
Country/region															
Albania	4,157	1.3	—	—	—	—	—	—	1,641	2.8	714	1.2	1,802	2.6	*χ* ^2^ (180) = 61,885.49; *p* < 0.001; *V* = 0.199
Austria	9,119	2.9	1,247	4.0	1,430	3.4	1,659	3.1	1,072	1.8	1,299	2.3	2,412	3.4	
Armenia	3,996	1.3	—	—	—	—	819	1.5	856	1.5	1,347	2.3	974	1.4	
Belgium (Flemish)	10,899	3.5	1,985	6.4	1,561	3.7	1,192	2.2	1,690	2.9	1,352	2.4	3,119	4.4	
Belgium (French)	4,944	1.6	444	1.4	627	1.5	446	0.8	576	1.0	1,349	2.3	1,502	2.1	
Bulgaria	3,963	1.3	—	—	—	—	—	—	1,562	2.7	1,484	2.6	917	1.3	
Canada	18,011	5.8	1,127	3.6	2,140	5.0	4,371	8.2	3,854	6.6	3,563	6.2	2,956	4.2	
Croatia	10,641	3.4	1,410	4.5	1,598	3.8	2,402	4.5	1,780	3.1	1,939	3.4	1,512	2.1	
Czechia	13,003	4.2	1,372	4.4	1,338	3.1	1,132	2.1	1,682	2.9	3,607	6.3	3,872	5.5	
Denmark	5,903	1.9	—	—	1,462	3.4	1,123	2.1	1,206	2.1	706	1.2	1,406	2.0	
Estonia	8,450	2.7	1,263	4.0	1,546	3.6	1,362	2.6	1,211	2.1	1,523	2.7	1,545	2.2	
Finland	8,985	2.9	1,720	5.5	1,594	3.7	2,035	3.8	1,855	3.2	982	1.7	799	1.1	
France	12,240	3.9	2,531	8.1	2,135	5.0	1,867	3.5	1,693	2.9	2,235	3.9	1,779	2.5	
Germany	9,479	3.0	366	1.2	2,376	5.6	1,540	2.9	1,927	3.3	1,430	2.5	1,840	2.6	
Greece	4,779	1.5	—	—	—	—	—	—	1,218	2.1	1,264	2.2	2,297	3.3	
Hungary	7,604	2.4	1,307	4.2	1,093	2.6	1,627	3.1	1,004	1.7	1,036	1.8	1,537	2.2	
Iceland	13,482	4.3	—	—	1,865	4.4	3,637	6.9	3,253	5.6	2,128	3.7	2,599	3.7	
Ireland	4,500	1.4	—	—	—	—	1,450	2.7	1,290	2.2	948	1.6	812	1.2	
Israel	6,510	2.1	1,165	3.7	796	1.9	676	1.3	769	1.3	1,657	2.9	1,447	2.1	
Italy	7,622	2.4	1,216	3.9	1,244	2.9	1,468	2.8	1,045	1.8	1,182	2.1	1,467	2.1	
Latvia	8,363	2.7	1,074	3.4	1,218	2.9	1,258	2.4	1,639	2.8	1,233	2.1	1,941	2.8	
Lithuania	9,975	3.2	1,888	6.1	1,824	4.3	1,718	3.2	1,665	2.9	1,120	1.9	1,760	2.5	
Luxembourg	6,446	2.1	—	—	1,439	3.4	1,299	2.5	1,023	1.8	1,285	2.2	1,400	2.0	
Malta	2,106	0.7	—	—	—	—	—	—	606	1.0	696	1.2	804	1.1	
Republic of Moldova	4,656	1.5	—	—	—	—	—	—	1,329	2.3	1,524	2.7	1,803	2.6	
Netherlands	8,025	2.6	1,266	4.1	1,346	3.2	1,404	2.6	1,293	2.2	1,458	2.5	1,258	1.8	
Poland	6,197	2.0	—	—	—	—	1,381	2.6	1,223	2.1	1,734	3.0	1,859	2.6	
Portugal	8,138	2.6	793	2.5	1,338	3.1	1,546	2.9	1,281	2.2	1,335	2.3	1,845	2.6	
Romania	8,933	2.9	—	—	1,527	3.6	1,929	3.6	1,393	2.4	1,477	2.6	2,607	3.7	
Slovenia	9,493	3.0	1,046	3.4	1,503	3.5	1,762	3.3	1,551	2.7	1,645	2.9	1,986	2.8	
Spain	7,254	2.3	—	—	—	—	1,348	2.5	3,189	5.5	1,432	2.5	1,285	1.8	
Sweden	10,133	3.2	1,196	3.8	1,480	3.5	2,018	3.8	2,660	4.6	1,539	2.7	1,240	1.8	
Switzerland	11,386	3.6	1,448	4.6	1,398	3.3	2,100	4.0	2,107	3.6	2,250	3.9	2,083	3.0	
North Macedonia	9,080	2.9	1,390	4.5	1,861	4.4	1,477	2.8	1,341	2.3	1,438	2.5	1,573	2.2	
England	7,380	2.4	1,693	5.4	1,401	3.3	1,051	2.0	1,519	2.6	711	1.2	1,005	1.4	
Scotland	9,198	2.9	1,124	3.6	2,064	4.9	2,317	4.4	1,673	2.9	1,274	2.2	746	1.1	
Wales	17,652	5.6	1,128	3.6	1,330	3.1	1,569	3.0	1,344	2.3	3,572	6.2	8,709	12.4	
Gender															
Boy	149,701	47.9	14,925	47.8	20,488	48.2	25,799	48.7	27,892	48.1	27,424	47.7	33,173	47.1	*χ* ^2^ (10) = 2,927.79; *p* < 0.001; *V* = 0.068
Girl	162,156	51.9	16,274	52.2	22,046	51.3	27,184	51.3	30,128	51.9	30,044	52.3	36,480	51.7	
No response	845	0.3	—	—	—	—	—	—	—	—	—	—	845	1.2	
Family affluence															
Lowest 20 percent	57,427	18.4	5,698	18.3	7,722	18.2	9,785	18.5	10,651	18.4	10,677	18.6	12,894	18.3	*χ* ^2^ (15) = 1,950.54; *p* < 0.001; *V* = 0.046
Medium 60 percent	185,114	59.2	18,812	60.3	26,056	61.3	30,795	58.1	32,830	56.6	34,529	60.1	42,092	59.7	
Highest 20 percent	60,472	19.3	6,238	20.0	7,905	18.6	11,139	21.0	11,299	19.5	10,500	18.3	13,391	19.0	
Missing	9,689	3.1	451	1.4	851	2.0	1,264	2.4	3,240	5.6	1,762	3.1	2,121	3.0	
Having at least one parental figure with whom communication is very easy															
None	178,767	57.2	19,499	62.5	26,234	61.7	30,362	57.3	32,564	56.1	31,187	54.3	38,921	55.2	*χ* ^2^ (10) = 3,666.27; *p* < 0.001; *V* = 0.077
At least one	127,184	40.7	11,268	36.1	15,072	35.4	20,119	38.0	24,669	42.5	25,483	44.3	30,573	43.4	
Missing	6,751	2.2	432	1.4	1,228	2.9	2,502	4.7	787	1.4	798	1.4	1,004	1.4	
Early first sexual intercourse															
Before 14	15,921	5.1	1,759	5.6	3,112	7.3	3,431	6.5	2,806	4.8	2,127	3.7	2,686	3.8	*χ* ^2^ (5) = 1,739.48; *p* < 0.001; *V* = 0.075
No intercourse, or at the age of 14+	296,781	94.9	29,440	94.4	39,422	92.7	49,552	93.5	55,214	95.2	55,341	96.3	67,812	96.2	

*Note*. — indicates that no data is available from the given country, the given survey year.

Weighted binary logistic regression models showing the changes in early intercourse from one survey year to another are presented in Supplementary Table S4 for the overall sample, Supplementary Table S5 for boys and Supplementary Table S6 for girls. The temporal pattern of change is summarised in Supplementary Table S7. In 14 countries, the odds of early initiation did not change, or decreased between two survey rounds. Only plateaus or increases between two rounds were seen in two countries. Odds of early initiation did not change across survey rounds, i.e., there was a stagnation, in five countries. The most prominent pattern, however, was a fluctuation over time. In other words, significant decreases, increases or no change between two survey rounds, varied in 16 countries. While in some countries the temporal pattern was similar for boys and girls, it was not uniform across all countries. We saw no clear geographical differences between the patterns.

### Multivariate Analysis: All Survey Rounds (2002–2022)

The sequence of the multivariate models is outlined in Table 1, Column (a). The results of models featuring those 24 countries that provided data for all six survey years are presented in Table 3. Survey year had a significant effect on early sexual intercourse; AIC and BIC indices reduced as the quadratic and cubic terms were introduced, indicating that the association between survey years and early sexual initiation can be best described by a cubic model. This result rhymes with the pattern in the Supplementary Tables that the change in the rates of early sexual intercourse in many countries did not happen in a linear fashion: overall, there was an increase from 2002 to 2006, a plateau from 2006 to 2010, and then continuous decrease from 2010 to 2022. Despite being significant, the odds ratios were very close to 1, indicating no uniform temporal change in early sexual initiation between 2002 and 2022. Introducing gender and family affluence further improved model fit: girls had significantly lower odds of reporting early sexual initiation compared to boys, and so did children from medium-affluent families compared to their peers from high-affluent families. Low family affluence also meant significantly lower odds, but that odds ratio (OR) was again very close to 1. Those who did not provide information on their family’s affluence had similar odds to early sexual initiation as those from high-affluent families.

**TABLE 3:** Multivariate analysis: factors influencing early sexual initiation in 24 countries, all survey years (Health Behaviour of School-aged Children study, 2002–2022).

	Model 5(a): Null models with cluster effect	Model 6(a):			Model 7(a): Gender and family affluence added	Model 8(a): Supportive parental figure added
		Year as linear term	Year as quadratic term	Year as cubic term		
		OR (SE)	OR (SE)	OR (SE)	OR (SE)	OR (SE)
Fixed effects						
Year, centred		0.969 (0.002)***	0.969 (0.002)***	0.934 (0.005)***	0.934 (0.005)***	0.933 (0.005)***
Year, centred (quadratic)			0.999 (0.0003)*	0.999 (0.0003)*	0.9993 (0.0003)*	0.9993 (0.0003)*
Year, centred (cubic)				1.0004 (0.0001)***	1.00005 (0.0001)***	1.0005 (0.0001)***
Gender (Girl)					0.585 (0.012)***	0.587 (0.012)***
Family affluence (reference: high)						
Medium					0.770 (0.020)***	0.768 (0.020)***
Low					0.925 (0.029)*	0.920 (0.030)**
Missing					1.210 (0.077)***	1.205 (0.080)**
Parental figure, very easy communication						1.075 (0.022)**
Random effects						
Variance explained by country (SE)	0.098 (0.029)	0.086 (0.026)	0.086 (0.026)	0.083 (0.025)	0.083 (0.025)	0.083 (0.025)
Variance explained by school (SE)	0.569 (0.026)	0.514 (0.025)	0.513 (0.025)	0.502 (0.025)	0.465 (0.024)	0.462 (0.024)
Intraclass correlation						
Country	0.025	0.022	0.022	0.022	0.022	0.022
School	0.169	0.154	0.154	0.151	0.143	0.142
Model fit						
AIC	85,095.26	84,841.62	84,837.45	84,784.79	83,968.59	83,958.86
BIC	85,126.30	84,883.01	84,889.19	84,846.87	84,072.06	84,072.68
Subsample sizes						
*n* (Participants)	230,205	230,205	230,205	230,205	230,205	230,205
*n* (Schools)	13,088	13,088	13,088	13,088	13,088	13,088
*n* (Countries)	24	24	24	24	24	24

Note. Model numbers are aligned with the respective step of the analytic strategy in Table 1. ***p < 0.001; **p < 0.01; *p < 0.05. OR, Odds Ratio; SE, Standard error; AIC, Akaike Information Criterion; BIC, Bayesian Information Criterion.

The addition of having a supportive parental figure further reduced AIC (while practically did not change BIC), implying that it increased the model fit. The respective odds ratio was significant, its value was very close to 1. In this model, 2.2% of the variance is explained by the difference between countries, and a much larger proportion (14.2%) by differences between schools.

### Multivariate Analysis: Last Three Survey Rounds (2014–2022)

The sequence of multivariate results featuring data from all 37 countries and the last three survey years are presented in Table 1, Column (b). Results are summarised in Table 4. In both 2018 and 2022, participants’ odds for early sexual initiation were significantly lower than in 2014, albeit they were not substantially lower than 0.9–0.8. Still, this was a robust pattern across the different models, adjusted for an increasing number of predictors. Adding gender and family affluence improved model fit: girls (contrasted to boys) and those from a medium- or low-affluent family (contrasted to those from high-affluent families) were significantly less likely to report early initiation, but again, the odds were not much different from 1. In the overall sample and among boys, those with a missing FAS response had significantly higher odds of reporting early initiation. Having a supportive parental figure did not make a practical difference in the odds of early intercourse, except an interaction between having a supportive parental figure × survey year (2022). However, this model (9b) had a higher BIC value than the one not including the interaction of parental support and survey years. Therefore, we returned to the previous model, 8(b) and applied it separately to boys and girls. The gender-disaggregated models are under column 10(b) in Table 4. Compared to 2014, there was already a small but significant decrease in the likelihood of early initiation (ORs between 0.7 and 0.9) for both boys and girls. From 2014 to 2022, the drop was significant in both genders. Family affluence was a significant predictor in both genders, but it only made a practical difference in girls’ early initiation (OR = 0.71 for medium and 0.78 for low affluence, respectively). For girls, a somewhat larger proportion of variation in early sexual intercourse was explained by school-related factors than for boys (22.2% and 14.8%, respectively); country differences had a lesser contribution, but again, they contributed more to the variation in girls’ early initiation than among boys (9.9% versus 3.0%, respectively).

**TABLE 4:** Multivariate analysis: factors influencing early sexual initiation in 37 countries, last three survey years (Health Behaviour of School-aged Children study, 2014–2022).

	Aggregate sample					Model 10(b): Stratified for gender	
	Model 5(b): Null model with cluster effect	Model 6(b): Year added	Model 7(b): Covariates added	Model 8(b): Adding supportive parental figure	Model 9(b): Adding supportive parental figure × survey year interaction	Boys	Girls
		OR (SE)	OR (SE)	OR (SE)	OR (SE)	OR (SE)	OR (SE)
Fixed effects							
Year (reference: 2014)							
2018		0.790 (0.030)***	0.803 (0.030)***	0.802 (0.030)***	0.788 (0.038)***	0.844 (0.038)***	0.703 (0.043)***
2022		0.820 (0.031)***	0.830 (0.031)***	0.830 (0.030)***	0.905 (0.041)*	0.792 (0.035)***	0.879 (0.051)*
Gender (Girl)			0.456 (0.012)***	0.457 (0.012)***	0.456 (0.012)***		
Family affluence (reference: high)							
Medium			0.705 (0.022)***	0.704 (0.022)***	0.704 (0.022)***	0.693 (0.027)***	0.710 (0.037)***
Low			0.967 (0.036) ns.	0.963 (0.036) ns.	0.964 (0.036) ns.	1.057 (0.050) ns.	0.779 (0.051)***
Missing			1.277 (0.080)***	1.275 (0.080)***	1.273 (0.080)***	1.394 (0.104)***	1.041 (0.122) ns.
Parental figure, very easy communication				1.043 (0.026) ns.	1.103 (0.045)*		
Parental figure × year (2018)					1.035 (0.064) ns.		
Parental figure × year (2022)					0.827 (0.048)**		
Random effects							
Variance explained by country (SE)	0.105 (0.026)	0.100 (0.025)	0.099 (0.025)	0.098 (0.025)	0.098 (0.024)	0.115 (0.030)	0.418 (0.109)
Variance explained by school (SE)	0.484 (0.030)	0.467 (0.030)	0.415 (0.028)	0.414 (0.028)	0.413 (0.028)	0.456 (0.039)	0.514 (0.061)
Intraclass correlation							
Country	0.027	0.026	0.026	0.026	0.026	0.030	0.099
School	0.152	0.147	0.135	0.135	0.135	0.148	0.220
Model fit							
AIC	60,041.11	60,001.28	58,822.03	58,821.22	58,809.31		
BIC	60,071.46	60,051.85	58,913.07	58,922.37	58,930.68		
Subsample sizes							
*n* (Participants)	182,574	182,574	182,574	182,574	182,574	86,880	95,694
*n* (Schools)	9,877	9,877	9,877	9,877	9,877	9,389	9,258
*n* (Countries)	37	37	37	37	37	37	37

Note. Model numbers are aligned with the respective step of the analytic strategy in Table 1. ***p < 0.001; **p < 0.01; *p < 0.05. OR, Odds Ratio; SE, Standard error; AIC, Akaike Information Criterion; BIC, Bayesian Information Criterion.

## Discussion

This paper investigated changes over time in the proportion of adolescents reporting early sexual intercourse. We explored variations in early intercourse across countries, and the role of gender, family affluence and parental support. We found that between 2002 and 2022, overall rates of early sexual initiation among adolescents from 37 countries slightly declined. Most changes occurred in the last three survey rounds (2014–2022). Exploring possible mechanisms behind these time changes was beyond the purpose of this paper, therefore we can only speculate on potential causes of the declining trend.

Given the cross-sectional design of the study repeating every 4 years with 15-year-olds, we believe that the changes in early sexual initiation combine a time and cohort effect [52]. In the first survey year, participants were born around 1987 (“Generation Y”); in the most recent one, around 2007 (“Generation Z”). The latter group is characterised as less prone to engage in adult activities such as having sex and drinking alcohol, which may be attributed to various reasons, including childhood lasting longer, changes in adolescents’ values and peer norms, and the spread of broadband internet and smartphones [53]. These may also have contributed to delaying the first sexual intercourse. It seems that the permeation of social media did not make young people more sexually active – on the contrary, it may have set the bar higher for offline romantic and sexual interactions. The fluctuating trends in some countries may also be associated with either introducing/facilitating or defunding/banning national sexual education curricula or sexual health promotion activities [54].

In the analyses featuring all survey rounds, the linear, quadratic and cubic terms were all significant, but they were close to 1, implying only a small change in the pooled data. This seems to be due to the large variation across countries in the temporal dynamics, which might have levelled each other out. In some countries, from one survey year to the next, alternating plateaus and decreases or increases were observed. In a small number of countries, we saw stagnation. In most countries, there was a fluctuation, but no clear geographical patterns were found. The 37 countries’ data in the last three survey years also varied. While we identified no directly comparable studies, these findings echo the variations observed in trends in reported sexual [21] and early sexual initiation [30].

In both arms of analysis (all versus the last three survey rounds), more variance was explained by school than by country differences. During the last three rounds, country accounted for 3% variance in boys’ early sexual initiation, while school factors explained 15%. On the other hand, country explained 10%, while school explained 22% in girls’ early sexual initiation. The impact of country variation is in line with the findings of de Graaf et al. [21], who saw that country differences explained 2% of its variance. Henderson et al. [55] argued that apart from schools’ differing levels of deprivation, other factors, such as peer groups, neighbourhood culture and youth-friendliness of the school may play a role here. Internationally, other school factors, including sexuality education and availability of condoms, may also contribute to the timing of sexual initiation [56]. Further studies need to disentangle the interaction between country- and school-level impacts and why country and school factors have a larger influence on girls’ than boys’ early sexual initiation.

Gender and family affluence were also significant contributors in the multivariate models: girls and youth from medium and (to a lesser extent) low affluent families were less likely, while those with a missing FAS response were more likely to report early sexual initiation. Girls in many cultures will be judged negatively if they have early sexual intercourse, while for boys, such behaviour is sometimes encouraged [57]. Other studies found mixed results regarding the association between SES and early sexual intercourse [12, 58, 59]. The decline in the last two survey years was evident in both boys and girls. We speculate that adolescents from low and medium socio-economic backgrounds might be more “guarded” against early intercourse than adolescents from high-affluent families. However, we acknowledge that this might be influenced by varying cultural norms across countries. Further studies are warranted to understand why participants with missing FAS responses reported higher rates of early initiation.

During the last three survey rounds, parental support did not have a practical effect in the gender-disaggregated sample independently. However, when analysed in conjunction with the 2022 survey round, it was found to significantly reduce the odds of early initiation (OR = 0.83). This indicates that the improvement in parental support between 2018 and 2022 had a protective role, which is a positive outcome. It seems that children’s sexual behaviour is more determined by communication with their parents nowadays compared to what it was two decades ago. Widman et al. [60] also found that better adolescent–parent communication was linked to safer sexual behaviour, and this effect was stronger for girls than boys; they also observed that these patterns were consistent in cross-sectional and longitudinal studies. Kushal et al. [26] found that globally, parental monitoring does, but parental support does not, protect against early sexual intercourse. We speculate that one reason for this could be that parental attitudes towards adolescent’s sexual activity largely vary across continents and countries – while our study had a much narrower geographical coverage.

### Strengths and Limitations

This study employed the rigorous HBSC methodology, including using representative samples of adolescents across all countries, standardised protocol, comparability of data, and including those participants who did not respond to the gender or FAS items. The study, however, has limitations. In some countries, for ethical reasons, it was not allowed to use certain responses (e.g., sexual intercourse under 13) or response categories were used outside of the standard range of those in the question (i.e., sexual intercourse at the age of 17 or older). The data were weighted in some, but not all, countries and survey years.

There were large differences in the subsample sizes. In some countries and genders, only a small proportion of the total sample reported sexual initiation. These factors resulted in an analytic sample with large differences in the subsample sizes across genders, countries and survey years, which may have reduced the statistical power and the reliability of some estimates.

We used a repeated cross-sectional design over a 20-year period. This means that the participants examined over time were not the same individuals. Therefore, time and cohort effects could not be separated. In the HBSC survey rounds before 2022, no standardised questions were used to assess birth-registered sex, gender identity and sexual orientation of adolescents, and such questions are still administered at the discretion of the national HBSC research teams.

There are other factors than family SES and parental support that may influence early sexual initiation, including onset of puberty, engagement in risk behaviours, exposure to pornography and relationships with peers, which were not included in the present analysis. These also show large cross-cultural variations. Future studies need to consider these factors as potential determinants of early sexual initiation.

### Policy and Practice Implications

Early sexual initiation is associated with negative sexual outcomes in later life, and it is often non-consensual [61]. Adolescents should be empowered to make an informed and autonomous decision on when (and with whom) they want to have their first sexual intercourse. Comprehensive sexuality education, covering assertiveness and efficient communication and teaching the importance of consent, is paramount in this regard. Meta-analytic evidence supports the efficacy of such educational programmes [62, 63]. Decision-makers should ensure that these standards of comprehensive sexuality education are implemented in national primary and secondary education internationally. Furthermore, considerations of gender, socio-economic status and other potential reasons for marginalisation (e.g., immigrant background, LGBTQ+ identity) must also be included in the development of such educational programmes. Our results also underline the protective role of having supportive parental figures, whom adolescents can trust. Empowering parents, teachers and youth workers with whom children in their care can comfortably and effectively discuss sexuality-related issues should also be prioritised [60, 64–66]. Given the large proportion of the variation in early sexual initiation explained by school differences, we believe that it should be ensured that comprehensive sexuality education is made mandatory on the national level, and teachers and schools get adequate support to integrate these classes or modules in their local curricula.

### Conclusion

Over the past two decades, there has been a slight but notable decline in the proportion of adolescents who have experienced sexual initiation before the age of 14. The rate of early initiation decreased from 6% in 2002 to 4% (3% among girls; 5% among boys) in 2022. Gender, family affluence and having a supportive parental figure also had an impact on the trends. While the proportion of youth engaging in early sex has decreased, given that early sexual initiation is associated with negative sexual health outcomes and is often non-consensual, concerted efforts are still needed from decision-makers, parents, teachers, and health and social care workers to help young people make informed decisions regarding their sexual initiation.
